# An Uncommon Cause of Cystic Lung Disease

**DOI:** 10.7759/cureus.114563

**Published:** 2026-08-15

**Authors:** Keegan Sorensen, Ali Khreisat, Mark Parker, Valentina Robila, Apostolos Perelas

**Affiliations:** 1 Internal Medicine, Virginia Commonwealth University, Richmond, USA; 2 Pulmonary and Critical Care Medicine, Virginia Commonwealth University, Richmond, USA; 3 Diagnostic Radiology, Thoracic Imaging Division, Virginia Commonwealth University, Richmond, USA; 4 Pathology, Virginia Commonwealth University, Richmond, USA

**Keywords:** amyloid cystic lung disease, cystic lesions of the lung, hypergammaglobulinemia, light chain deposition disease, sjögren’s syndrome

## Abstract

A 59-year-old female with Sjögren's syndrome presented with a three-year history of dyspnea and cough beginning after COVID-19 infection. Serial chest computed tomography scans over a course of three years revealed multiple pulmonary cysts and nodules, including one that enlarged over time. Pulmonary function was largely preserved, aside from a mildly reduced diffusing capacity, while laboratory studies showed hypergammaglobulinemia and elevated kappa light chains. After a nondiagnostic bronchoscopic transbronchial biopsy, she underwent thoracoscopic wedge resection. Pathology revealed eosinophilic deposits without Congo red staining, and mass spectrometry confirmed light chain deposition disease. Bone marrow biopsy excluded malignancy, and treatment with mycophenolate led to symptomatic improvement. This case highlights the diagnostic challenges often encountered with an uncommon cause of cystic lung disease.

## Introduction

Cystic lung diseases comprise a heterogeneous group of disorders characterized by air-filled spaces with thin, imperceptible walls in the lung parenchyma. The differential diagnosis includes more commonly recognized entities, such as lymphangioleiomyomatosis, pulmonary Langerhans cell histiocytosis, and lymphocytic interstitial pneumonia, as well as less frequent, often underrecognized conditions [1]. In clinical practice, affected patients often present with nonspecific respiratory symptoms, and radiologic imaging findings may overlap considerably, limiting the specificity of imaging alone [2].

Although high-resolution computed tomography (HRCT) has improved the characterization of cystic patterns, establishing a definitive diagnosis frequently requires correlation with clinical history, serologic data, and, in select cases, histopathologic evaluation [3]. This is particularly relevant in those individuals with autoimmune conditions such as Sjögren's syndrome, where multiple etiologies for cystic lung changes may coexist. Consequently, diagnostic uncertainty is not uncommon, and a multidisciplinary approach is often necessary. This case underscores the diagnostic challenges inherent in evaluating cystic lung disease and the importance of thorough, integrative, and collaborative assessment [4,5].

## Case presentation

**Figure 1:**
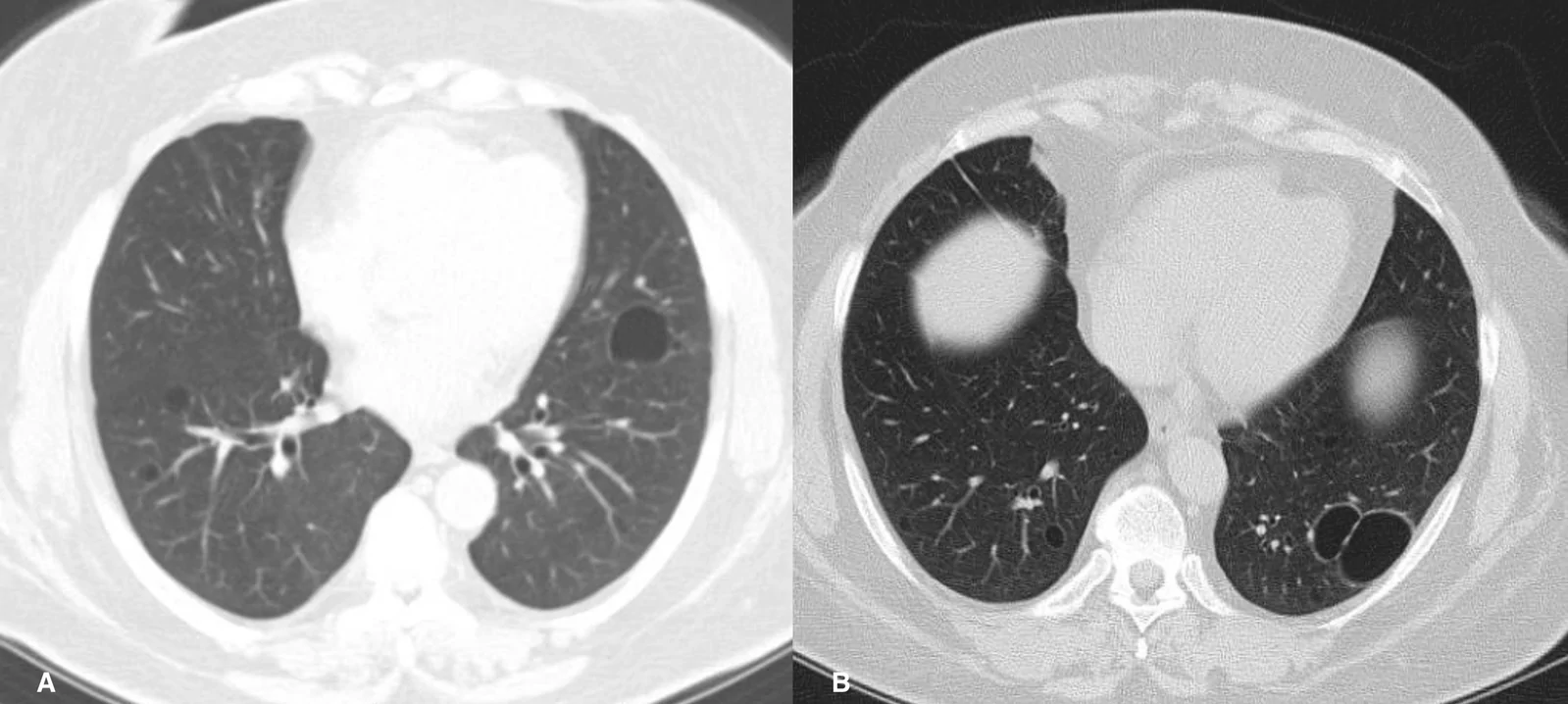
(A) CT of the chest showing variable-sized pulmonary cysts in the right and left lower lobes. (B) Interval increase in the left lower lobe cyst, along with scattered calcified pulmonary nodules.

The patient was a 59-year-old female with a past medical history of mild intermittent asthma, hypertension, hypothyroidism, and Sjögren's syndrome. She was referred to the pulmonary clinic for a second opinion for evaluation of dyspnea and dry cough. Her symptoms had persisted for approximately three years following a COVID-19 infection. She was a lifelong nonsmoker and employed as a billing specialist working from home. During her workup, she underwent three sequential chest CTs that showed multiple, variable-sized, thin-walled pulmonary cysts and non-calcified as well as calcified, non-cavitary, solid nodules (Figure 1A). One cyst demonstrated interval enlargement on serial imaging (Figure 1B). Selected laboratory findings are summarized in Table 1. Pulmonary function testing demonstrated preserved spirometry (Table 2), including a forced vital capacity (FVC) of 3.21 L, a forced expiratory volume in one second (FEV₁) of 2.70 L, and an FEV₁/FVC ratio of 84%. The uncorrected diffusing capacity for carbon monoxide (DLCO) was mildly reduced at 15.53 mL/min/mmHg (70% predicted; Z score, −2.15).

**Table 1:** Laboratory evaluation.

Laboratory test	Patient's result	Reference range/Interpretation
C-reactive protein (CRP)	0.5 mg/dL	<0.8 mg/dL
Erythrocyte sedimentation rate (ESR)	94 mm/hr	0-30 mm/hr
Serum protein electrophoresis (SPEP)	Polyclonal hypergammaglobulinemia	Normal electrophoretic pattern
Serum immunofixation electrophoresis (IFE)	No monoclonal protein detected	Negative
Urine protein electrophoresis (UPEP)	No monoclonal protein detected	Negative
IgG	3,046 mg/dL	700-1,600 mg/dL
Kappa free light chain	46.81 mg/L	3.3-19.4 mg/L
Lambda free light chain	21.31 mg/L	5.7-26.3 mg/L
Kappa/Lambda ratio	2.20	0.26-1.65
Anti-SSA (Ro) antibody	>8 AI	<1.0 AI (negative)
Anti-SSB (La) antibody	>9 AI	<1.0 AI (negative)
B-type natriuretic peptide (BNP)	10 pg/mL	<100 pg/mL
Vascular endothelial growth factor-D (VEGF-D)	314 pg/mL	Within institutional reference range

**Table 2:** Pulmonary function testing.

Pulmonary function parameter	Patient's result	% Predicted	Z score
Forced vital capacity (FVC)	3.21 L	90%	−0.59
Forced expiratory volume in 1 second (FEV₁)	2.70 L	96%	−0.20
FEV₁/FVC ratio	84%	106%	+0.89
Uncorrected diffusing capacity for carbon monoxide (DLCO)	15.53 mL/min/mmHg	70%	−2.15
Alveolar volume (VA)	4.52 L	83%	−1.44
Transfer coefficient for carbon monoxide (KCO, DLCO/VA)	3.43 mL/min/mmHg/L	84%	−1.06

**Figure 2:**
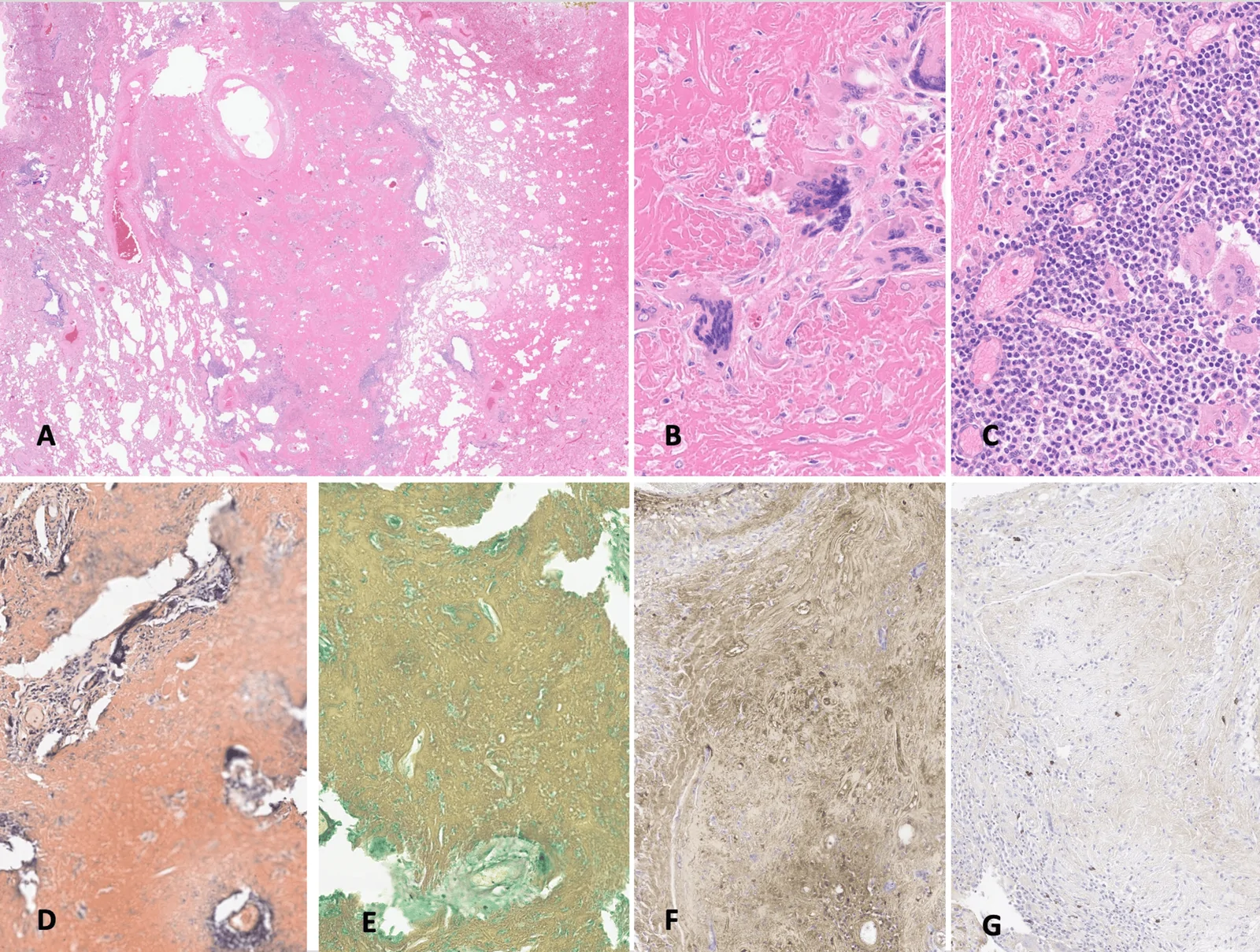
Histopathological findings of light chain deposition disease (LCDD). (A) Nodular area with central amorphous eosinophilic material and a rim of inflammatory cells on hematoxylin and eosin stain (2x). (B) The amorphous material has embedded multinucleated giant cells (40x). (C) The rim of the nodule contains lymphoplasmacytic inflammation and scattered giant cells (40x). (D) Congo red demonstrates nonspecific orange-red staining without apple-green birefringence under polarized light. (E) Sulfonated Alcian blue stains light pink-tan (10x). (F) Immunohistochemical stains (20x) show deposition of immunoglobulin kappa chains. (G) No deposition of lambda chains on immunohistochemical stain (20x).

She received a prednisone burst, which improved both her dyspnea and cough but did not affect the size of the pulmonary nodules. A positron emission tomography/computed tomography (PET/CT) (not illustrated) revealed borderline increased uptake in the dominant lung nodule and no evidence of hypermetabolic mediastinal adenopathy. An attempted sampling of the dominant nodule using navigational bronchoscopy fine needle aspiration was nondiagnostic. She subsequently underwent a right lower lobe wedge resection. The surgical pathology revealed an accumulation of amorphous, eosinophilic material along with an incidental carcinoid tumorlet (Figure 2). There was no histopathologic or radiologic evidence of any neuroendocrine proliferative process. The sample was sent to a reference laboratory for further analysis. Congo red demonstrated nonspecific orange-red staining but lacked apple-green birefringence under polarized light, excluding amyloid deposition. Liquid chromatography-tandem mass spectrometry (LC-MS/MS) performed on laser microdissected tissue demonstrated abundant κ immunoglobulin light chain peptides without amyloid-associated proteins (including serum amyloid P component, apolipoprotein A-IV, or apolipoprotein E), confirming non-amyloid light chain deposition disease (LCDD). The case was subsequently discussed in our multidisciplinary interstitial lung disease conference, and a consensus diagnosis of LCDD was reached.

She was subsequently evaluated by hematology, and bone marrow biopsy demonstrated a cellular marrow with maturing trilineage hematopoiesis and a mild increase in polytypic plasma cells (5-7%) without evidence of a plasma cell dyscrasia. She was started on mycophenolic acid (Myfortic) 360 mg twice daily as a steroid-sparing agent for Sjögren's syndrome. At 12-month clinical and radiographic follow-up, she reported improvement in dyspnea, and repeat chest CT demonstrated stable bilateral pulmonary cysts without progression.

## Discussion

Sjögren's syndrome is an autoimmune condition associated with lymphocyte infiltration of glandular tissues, namely, the lacrimal and salivary glands. Extraglandular involvement is also common [1]. Pulmonary manifestations vary and can include bronchiectasis, interstitial lung disease, and chronic bronchiolitis. The most common symptoms are cough and dyspnea [1,2]. Chest CT findings include consolidation, septal and interstitial thickening, ground-glass opacities, and cysts [4,5]. Current evidence suggests that pulmonary cyst formation in LCDD results from deposition of monoclonal light chains within small airway basement membranes, producing partial bronchiolar obstruction and chronic air trapping. Concurrent disruption of alveolar supporting structures and matrix metalloproteinase-mediated extracellular matrix degradation further weakens the lung parenchyma, leading to progressive enlargement of thin-walled cysts [6].

A rarely reported pulmonary manifestation of Sjögren's syndrome is LCDD [7,8]. In addition to Sjögren's syndrome, this non-amyloid monoclonal gammopathy has been associated with lymphoproliferative disorders such as multiple myeloma and lymphoma [8,9]. The most common site of deposition is the kidney, with hepatic, cardiac, and pulmonary involvement less common [9]. Two forms of LCDD, nodular and cystic, have been described based on imaging. Although often described as distinct radiologic phenotypes, these patterns may coexist [10]. Our patient demonstrated both cystic lung disease and pulmonary nodules, illustrating the overlap between the two recognized radiographic phenotypes of pulmonary LCDD.

This case highlights the importance of histopathology in confirming a definitive diagnosis. A variety of cystic lung conditions have been reported in patients with Sjögren's syndrome, including lymphocytic interstitial pneumonia (LIP) and amyloidosis. Moreover, the patient’s age and gender placed her at risk for other cystic lung conditions such as lymphangioleiomyomatosis. A high degree of clinical suspicion is necessary for accurate diagnosis, specifically as the imaging findings are often non-specific and the tissue may lack pathognomonic features.

Patients with LCDD may report symptoms such as dyspnea, cough, or fatigue, but a significant proportion remain asymptomatic, especially when the disease is not extensive. In this case, our patient remained symptomatic after completing pulmonary rehabilitation; thus, we determined that a therapeutic trial was warranted.

There are no current FDA-approved treatments for LCDD. The goal of therapy is to decrease the production of light chains and, therefore, minimize organ damage. Treatments targeting multiple myeloma have been used for patients with both LCDD and multiple myeloma, including anti-CD38 monoclonal antibody treatment [9]. For patients without concurrent lymphoproliferative disorders, hematopoietic stem cell transplant and varied chemotherapy regimens, including protease inhibitors and alkylating agents, have been used [9,11]. Data regarding treatment specifically for pulmonary LCDD remain limited. Lung transplantation may be needed in cases of progressive respiratory failure [11]. In this case, the patient experienced symptomatic improvement, and imaging remained radiographically stable over 12 months following initiation of mycophenolic acid. Although this temporal association is encouraging, no conclusions regarding treatment efficacy can be drawn from a single case.

The long-term clinical course and survival for patients with pulmonary LCDD is not well described. A retrospective review of 10 cases of nodular pulmonary LCDD showed prognosis was generally favorable at a median two- to three-year clinical follow-up, with most patients not receiving treatment [7]. A retrospective study looking at cystic pulmonary LCDD showed a median annual decline in FEV₁ of 127 ml/year and a decline in diffusing capacity (DLCO) of 4.8%/year. Three patients in this study died from progressive respiratory failure [11].

## Conclusions

This case highlights the diagnostic challenges of pulmonary LCDD, a rare and often underrecognized cause of cystic lung disease in patients with Sjögren's syndrome. As imaging and clinical findings can overlap with more common conditions, a definitive diagnosis requires multidisciplinary evaluation and often surgical biopsy. Following initiation of mycophenolic acid, the patient experienced symptomatic improvement and radiographic stability over 12 months of follow-up. Although encouraging, this temporal association does not establish treatment efficacy for pulmonary LCDD. Further studies are needed to better characterize the natural history of pulmonary LCDD and to evaluate optimal management strategies for this rare disease.
